# High Concentration Crystalline Silk Fibroin Solution for Silk-Based Materials

**DOI:** 10.3390/ma15196930

**Published:** 2022-10-06

**Authors:** Danyu Yao, Ting Wang, Xiaoli Zhang, Yuqing Wang

**Affiliations:** 1 School of Automation, Hangzhou Dianzi University, Hangzhou 310018, China; 2 School of Physics, Huazhong University of Science and Technology, Wuhan 430074, China; 3 Key Laboratory for Biomechanics and Mechanobiology of Ministry of Education, Beijing Advanced Innovation Center for Biomedical Engineering, School of Biological Science and Medical Engineering, Beihang University, Beijing 100083, China

**Keywords:** crystalline silk fibroin solution, high concentration, hydrogel, sonication

## Abstract

As a functional biomaterial, silk fibroin has been widely used in drug release, cell encapsulation and tissue regeneration. To meet the requirements of these applications, the properties of silk fibroin-based materials should be finely tunable. Many useful properties of biomaterials emerge from the collective interactions among ordered and disordered domains. Thus, increasing subtle control of silk hierarchical structures is required. As a characteristic of ordered silk fibroin, crystalline silk fibroin (CSF) is an important part of silk fibroin-based biomaterials, but the preparation of CSF solution, especially high concentration CSF solution, remains a challenge. Here, a solution composed of β-sheet-rich silk fibroin is reported. These CSF were obtained by the sonication of silk fibroin hydrogel, destroying the hydrogel network, and turning silk fibroin hydrogels into CSF solution. These β-sheet-rich CSF solutions were stable enough for several days or even weeks. In addition, they were typically ordered crystalline domains, which could be mixed with disordered domains and fabricated into porous scaffolds, films, hydrogels and other silk fibroin-based scaffolds with different properties.

## 1. Introduction

As a natural protein, silk fibroin has many impressive properties, such as low immunogenicity, good biocompatibility, excellent elasticity and strength [1,2,3]. It has been widely used in tissue repair [4], drug release [5] and other biomedicine applications in the form of hydrogels [6,7], electrospun [8,9], porous scaffolds [10,11], etc. Each application has different requirements for material properties, which requires the flexible regulation of the properties of silk fibroin-based biomaterials [12,13]. Studies indicated that many useful properties of polymeric materials emerge from the collective interactions among ordered and disordered domains [14]. Typically, the extraordinary combination of elasticity and strength of nature silk fibers should be the result of the interactions among periodically repeated β-sheets and unstructured domains encoded by the primary sequence [15]. To tune the properties of regenerated silk fibroin-based materials, subtle control of silk hierarchical structures with ordered or disordered domains in aqueous solution is required. This, however, remains a challenge.

There are many reports on the preparation of amorphous silk fibroin solution [16,17,18]. The regeneration of silk fibroin began from the degumming of silk fibers after which the fibers were dissolved in different solvents, such as inorganic salts [16], concentrated acids [17], and ionic liquids [18]. Extensive hydrogen-bonds among silk fibroin molecules were destroyed, resulting in amorphous silk fibroin solution. However, there are few reports on the preparation of well-dispersed crystalline silk fibroin (CSF) aqueous solution, especially the high concentration CSF aqueous solutions. CSF solution could be obtained by mixing a large amount of alcohol with low concentration (<0.75%) silk fibroin solution in a short time [19]. Silk fibroin molecules could be assembled into elongated nanofibrils; however, the use of alcohol was not conducive to subsequent cell culture and tissue regeneration. In another study [20], regenerated ordered silk fibroin composed of silk nanofibers with a β-sheet structure about 10−20 nm in diameter in aqueous solution via a complex thermodynamically driven process has been reported. Silk fibroin maintained a solution state at low concentrations due to the high charge repulsion and high beta-sheet content; however, it transitioned to the hydrogel state at higher concentrations (>0.5%), limiting its use for many silk fibroin-based applications. The preparation of CSF solution needs to be further explored.

Recently, we found that all-aqueous silk hydrogel can be fabricated through silk fibroin molecules’ self-assembly [21]. Ultrasonic treatment has been widely used to fabricate small clusters or individual nanoparticles [22]. In the present study, we show that such a hydrogel can be further converted to a high concentration CSF solution through a simple ultrasonic treatment process. The preparation process of the solution didn’t need to add any organic solvents, which retained the good biocompatibility of silk fibroin, and provided a good foundation for the application of silk fibroin-based biomaterials.

## 2. Materials and Methods

The Bombyx mori silkworm fibers were obtained from China (Jiuyuan mulberry silk, Hangzhou, China). For silk degumming, Na_2_CO_3_ (Sigma-Aldrich, St. Louis, MO, USA) was used. LiBr (Sigma-Aldrich, St. Louis, MO, USA) was used to dissolve the degummed silk fibers, and cellulose membranes (3500 MWCO, Thermo Scientific, Rockford, IL, USA) were used to dialyze the solution. Gelatin (Sinopharm, Peking, China) solution was used to mix with CSF and form hydrogel, chondrocytes (YaJi Biological, Shanghai, China) were cultured with the mixture of DMEM-F12 (Meilunbio, Dalian, China), fetal bovine serum (Sangon Biotech, Shanghai, China), and antibiotic/antimycotic (Sangon Biotech, Shanghai, China). The hydrogels with encapsulated cells were stained with a live/dead Viability Kit (Life Technologies, Grand Island, NY, USA).

### 2.1. Preparation of Crystal Silk Fibroin (CSF) Solution and CSF-Based Biomaterials

#### 2.1.1. Fabrication of Fresh Silk Fibroin Solution

The fresh silk fibroin (SF) aqueous solution was fabricated as previously reported [23]. Briefly, 5 g *B. mori* silkworm fibers were treated with boiling 0.02 M Na_2_CO_3_ solution for 30 min and washed with deionized water to thoroughly remove the sericin on the fiber surface. The left silk fibroin fibers were air-dried before being solubilized in 9.3 M LiBr at 60 °C for 4 h. It was then dialyzed against distilled water with a regenerated cellulose membrane for three days. Finally, the solution was centrifuged with a centrifuge (Cence, TG16-WS, Changsha, China) at 9000 rpm for 20 min at 4 °C to remove the insoluble particulates. One mL silk fibroin solution was taken out and weighed, dried and weighed again, and its mass fraction was calculated.

#### 2.1.2. Preparation of CSF Solution

The SF solution was concentrated to over 25 wt% within three days in a fume hood as previously described [21,24]. The solution was cast into polystyrene Petri dishes (diameter 100 mm) and covered with lids with different numbers of holes to control the concentrating rates. The concentrated solution was diluted to 1–5 wt% with distilled water and incubated at 60 °C overnight to induce nanofiber formation. Subsequent to this, it was ultrasonically treated with a SL-650D Sonifer (Shunliu Instrument Co., Nanjing, China) for 1 min with an output power supply of 30%, which led to the generation of the crystal silk fibroin (CSF) solution. The CSF solution was further ultrasonically treated to study the effect of ultrasonic treatment on CSF viscosity, and the treated solution is called ultrasonic treatment CSF solution.

#### 2.1.3. Preparation of CSF-Based Hydrogel

The CSF solution and fresh SF/gelatin solution were mixed, and the hydrogels formed after several minutes.

#### 2.1.4. Preparation of CSF-Based Films

To prepare silk fibroin films, 1 mL CSF solution was casted on polystyrene Petri dishes (diameter 35 mm) and dried overnight at room temperature.

#### 2.1.5. Preparation of CSF-Based Porous Scaffolds

Fresh SF solution and CSF solution was mixed and poured into cylindrically-shaped containers. They were then placed at −80 °C to freeze the samples and the frozen samples were lyophilized for about 48 h to achieve porous scaffolds.

#### 2.1.6. Cell Culture

The DMEM-F12 supplemented with 10% fetal bovine serum and 1% antibiotic/antimycotic were used to culture the chondrocytes. All solutions were sterilized with a low temperature intermittent sterilization process before they were used to fabricate scaffolds. Briefly, solutions were placed into an oven at 60–80 °C for 1 h and then took them out and kept at 37 °C for 24 h, and this process was cycled three times. Cell suspension was mixed with fresh SF aqueous solution before they were mixed with CSF solution, and the final cell concentration was 10^4^ mL^−1^. Before hydrogel formation, 100 µL per well was added to 48 well plates and then the plates were placed into an incubator maintained at 37 °C and 5% CO_2_ for 1H for gelation. Finally, 200 µL media was added per well.

### 2.2. Characterization

#### 2.2.1. Scanning Electron Microscopy (SEM)

The hydrogel samples were lyophilized and cut into slices with a thickness of 1–2 mm. The samples were then pasted on the test bench with conductive adhesive. The morphology of the cross-section of the samples were examined by SEM (S-4800, Hitachi, Tokyo, Japan). For silk fibroin solution samples, the solution was diluted with deionized water and then two microliters of protein samples were dropped onto a clean silicon wafer. The samples were dried in air and then coated with gold before being examined by SEM.

#### 2.2.2. Fourier Transform Infrared Spectroscopy (FTIR)

The secondary structure of samples was analyzed with a FTIR spectrophotometer (Thermo Scientific, Miami, FL, USA). The scaffolds were lyophilized and cut into slices with a thickness of 1–2 mm. For each test, 64 scans were coded with a resolution of 4 cm^−1^, with the wavenumber ranging from 4000 to 400 cm^−1^.

#### 2.2.3. Circular Dichroism (CD)

The secondary structures of silk fibroin solutions were measured with an Aviv model 62DS spectrophotometer equipped with a temperature controller (AVIV Biomedical, Inc., Lakewood, NJ, USA). The CD spectra were recorded from 260 to 190 nm at a resolution of 0.5 nm.

#### 2.2.4. Viscosity

Rheological studies were applied on solutions to show the effect of ultrasonic treatment on the viscosity status of the CSF solution. All rheology was carried out on an Anton Paar MCR52 rheometer (Anton Paar, Shanghai, China). The viscosity of silk fibroin solution was collected over the shear rate range of 100 to 1000 s^−1^.

#### 2.2.5. Live/Dead Viability

The encapsulated cells were stained with a live/dead Viability Kit according to the manufacturer’s instructions. Briefly, Calcein AM and PI solutions were diluted to the specified concentration, and then mixed with the sample according to the instructions, followed by 30 min’s of incubation to stain live (green) and dead cells (red), respectively. The samples were observed by a fluorescence microscope (Leica, Germany) after staining.

### 2.3. Statistical Methods

All statistical analyses were performed using SPSS v.16.0 (IBM Corp., Armonk, NY, USA). Comparison of the mean values of the data sets was performed using one-way AVOVA. Measures are presented as means ± standard deviations, unless otherwise specified. *p* < 0.05 was considered significant.

## 3. Results and Discussion

### 3.1. Microstructure of Crystal Silk Fibroin

**Figure 1:**
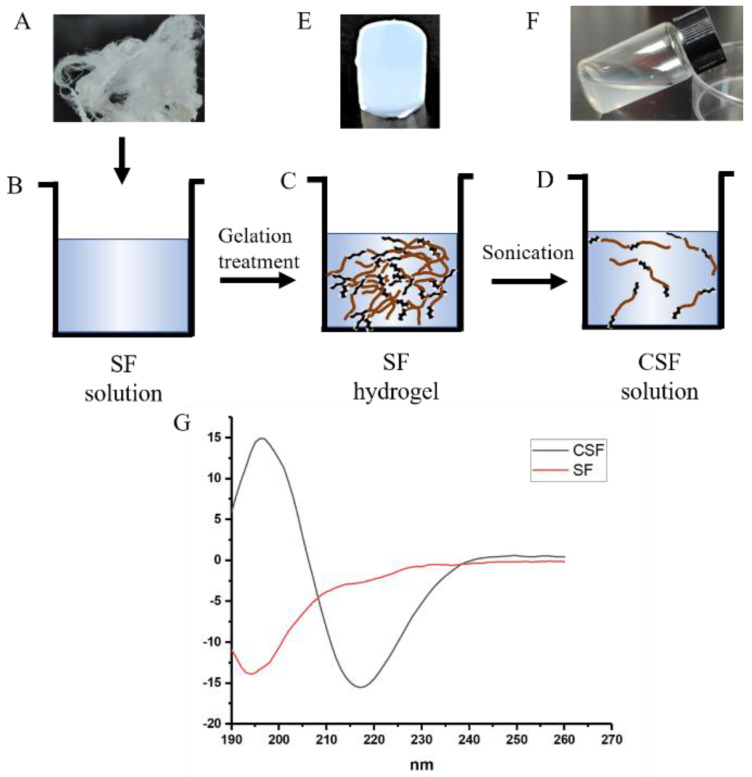
Scheme of the strategy to prepare CSF solution. Degummed silk fiber (**A**) was dissolved to form fresh silk fibroin solution (**B**), and then the solution was gelation treated to obtain silk fibroin hydrogel (**C**). Finally, the hydrogel was ultrasound treated, and this led to the formation of a CSF solution (**D**). (**E**,**F**): the morphology of silk fibroin hydrogel (**E**) and the CSF solution (**F**); (**G**): CD curves of fresh silk fibroin solution and the CSF solution.

The preparation of CSF solution is shown in Figure 1. First, fresh SF solution was obtained with the reported method. It was then gelation treated and formed a hydrogel, during which the random coil structure silk fibroin was transformed into a stable crystalline structure. Finally, the hydrogel was ultrasound treated, which broke the hydrogel network and led to the formation of a flowable solution.

It has been proven that ultrasonication is a typical way to induce the formation of silk fibroin hydrogels [25]. Sonication induces changes in hydrophobic hydration which results in the accelerated formation of β-sheet crystals. After the ultrasonic treatment, the initial rapid formation of β-sheet structure occurred, and the exposure of hydrophobic domains of β-sheet crystals promoted the intermolecular hydrophobic interactions between random coil molecules, thus promoting the transformation of a silk fibroin structure from the amorphous random coil to a crystalline β-sheet structure. The formation of β-sheet crystals and the subsequent physical cross-links resulted in the formation of a hydrogel network. Interestingly, when silk fibroin hydrogel was treated by ultrasound, we obtained a flowing solution (Figure 1A–F). The secondary structure of silk fibroin in these solutions were measured by CD test (Figure 1G); a strong negative peak appeared at 195–202 nm in the curve of the fresh SF solution, indicating random coil structure, while the solution from the hydrogel displayed a strong positive peak at 185–200 nm, indicating β-sheet structure.

The concentration of silk fibroin hydrogel had a key effect on the formation of CSF solution. Under the same ultrasonic power, we got different results according to the initial silk fibroin concentration (Figure S1). When silk concentration was below 5%, the hydrogels could be dispersed into flowable solutions (Figure S1A). When the concentration of silk fibroin increased to 6%, the morphology of hydrogels was destroyed after ultrasonic, but remained solid state instead of being converted to solution (Figure S1B). Ultrasonic power was another key factor in the formation of CSF solution (Figure S2). When the 5% hydrogel was treated with different ultrasonic power, only a small amount of hydrogel was converted into solution when the ultrasonic power was 10%. With the increase of ultrasonic power, the conversion efficiency increased gradually and reached the highest when the power was 70%. According to previous studies, in sonication, mechanical vibration causes the formation and collapse of bubbles [26]. As a result of this cavitation, the media may experience extreme local effects: heating (10,000 K), high pressure (200 bar) and high strain rates (10^7^ s^−1^) [27]. High pressure and high strain rates might destroy the hydrogel network and led to the formation of CSF solution, while heating promoted the self-assembly of silk fibroin [24], which was conducive to the formation of hydrogels. Thus, the proper enhancement of ultrasonic power was conducive to improving the efficiency of hydrogel solution conversion. However, excessive power might cause the rapid rise of the temperature of the treated area, which was not conducive to the formation of the solution.

**Figure 2:**
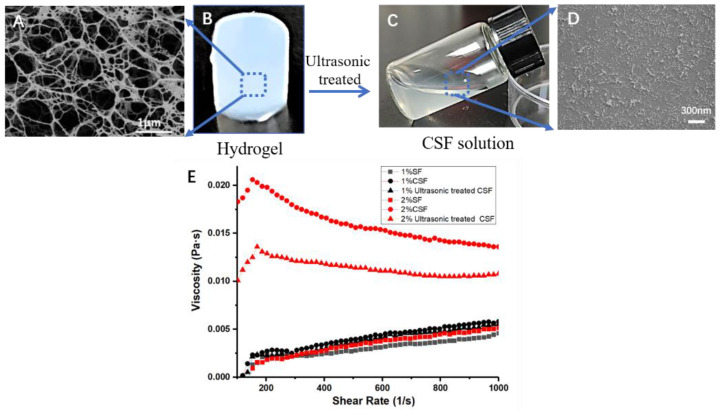
Morphology of silk fibroin in hydrogel (**A**,**B**) & CSF solution (**C**,**D**) and the viscosity of solutions (**E**).

The morphology of the hydrogel before ultrasonic treatment and the solution produced by it were observed through SEM (Figure 2). The hydrogels (Figure 2A) were freeze-dried to retain the morphological structure of the proteins, and then observed by SEM (Figure 2B). In the hydrogel, silk fibroin was aggregated into a three-dimensional fibrous network structure, forming the skeleton of the hydrogel. After ultrasonic treatment, the hydrogel turned into CSF solution (Figure 2C). And in the CSF solution, silk fibroin was dispersed into particles and short nanofibers ranging from tens to hundreds of nanometers (Figure 2D). The energy provided by ultrasound promoted the breakage of skeleton of the hydrogel net and silk fibroin, which originally had a three-dimensional network connection structure, was dispersed into fragments to form particles and nanofibers.

The flowability of CSF solution and the effect of ultrasonic treatment on the viscosity status of CSF solution was characterized by rheological measurements (Figure 2E). The viscosity of all solutions was very low (<0.025 Pa·s), which endowed them with good flowability. Fresh silk fibroin solution would be transformed into hydrogel after ultrasonic treatment [25]. In contrast, the CSF solution remained in a solution state after ultrasonic treatment because silk fibroin in the CSF solution had formed a stable crystalline structure (Figure 1G). The viscosity of both 1% and 2% CSF solution decreased slightly after ultrasonic treatment. This result was consistent with previous studies. Ultrasonic treatment could break long nanofibers into shorter fibers [20], or disperse large aggregates into small particles [22], resulting in a decrease in solution viscosity.

According to the SEM morphology of hydrogels (Figure S3), silk fibroin in the gel formed fiber-like aggregations and the density of the aggregations increased significantly with the increase of silk fibroin. The mechanical strength of hydrogels increased with the increase of silk fibroin concentration [28], which indicated that the links between silk fibroin in the hydrogel were enhanced, which made it difficult to disperse silk fibroin. Ultrasonication could break weak networks and resulted in fibrous silk fibroin fragments (Figure 2). However, when the density of aggregations was big enough, the entanglement between silk fibroin segments was tighter and more difficult to be dispersed, and it thus remained in the hydrogel state. Therefore, low concentration hydrogels were easily dispersed, while high concentration hydrogels were difficult to be dispersed into solutions. On the other hand, increasing ultrasonic power could increase the output per unit time, which was conducive to the destruction of the hydrogel network and dispersing it into solution. However, at the same time, the dense hydrogel network also made it difficult to transmit ultrasonic energy. When the ultrasonic power was too large, it could generate a lot of heat in a short time, which led to the sharp rise of temperature of the treated parts, and the high temperature promoted the conversion of silk fibroin solution into hydrogel. Under the combined action of two factors, the hydrogel of low concentration could be converted into solution under the action of ultrasound, while the high concentration gel will only be fragmented and difficult to disperse.

It is worth noting that reported methods to fabricate CSF can only prepare silk fibroin solution with concentration less than 1%, which greatly limits the application of silk fibroin materials, while the method described in this work can directly obtain the CSF solution with a concentration of up to 5%, which provides a great platform for the construction of silk fibroin-based biomaterials. Moreover, the whole preparation process does not need any cytotoxic additives, and the operation is simple and convenient, which is very conducive to the preparation and application of silk fibroin-based materials.

### 3.2. Structural Analysis

**Figure 3:**
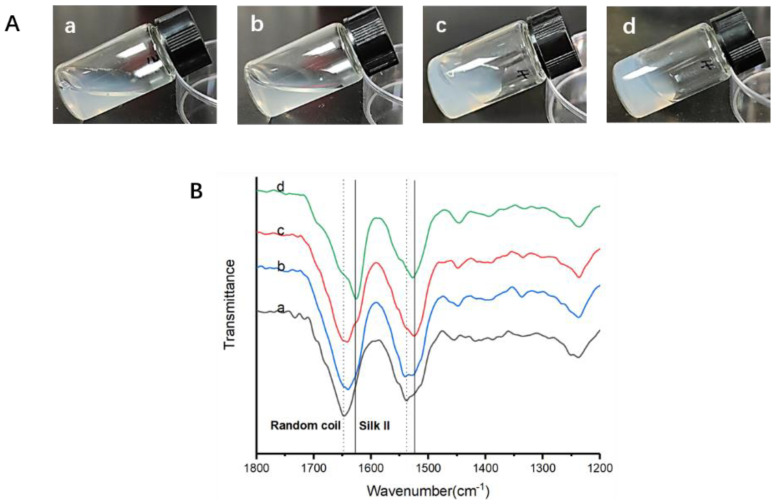
The morphology (**A**) and FTIR results (**B**) from solution to hydrogel. a. Fresh silk fibroin (SF); b. Mixed with crystal silk nanofiber solution; c. Flowable hydrogel; d. hydrogel.

The preparation of CSF solution had two key processes: the formation of the silk fibroin network and the redispersion of silk fibroin under the action of ultrasound. The structural changes f during this process were determined by FTIR. During the formation of the hydrogel network, silk fibroin underwent a significant structural change (Figure 3). The FTIR spectral region within 1700–1600 cm^−1^ (amide I) and 1600–1500 cm^−1^ (amide II) has been commonly used for the analysis of the secondary structure of silk fibroin. The peaks at 1520–1540 cm^−1^ are characteristic of random coil structures, while the peaks at 1610–1630 cm^−1^ and 1510–1520 cm^−1^ indicate the silk II’s secondary structure [29]. The fresh silk fibroin showed a peak at 1648 cm^−1^, indicating a random coil structure. After being mixed with crystal silk nanofiber solution, the SF solution turned into a flowable hydrogel and then formed a stable hydrogel. The peak at 1648 cm^−1^ gradually decreased and a new peak formed at 1627 cm^−1^, indicating the silk II structure.

Deconvolution of the infrared spectral region in the amide I region was performed by Peakfit to assess the contents of different secondary structures (Table 1). The content of β-sheet, the most ordered crystalline structure, increased from 21.9 ± 0.5 to 48.4 ± 0.3 percent. The a-helix and turns/bends are intermediate states before silk II (mainly β-sheet) formation. The content of amorphous random coil structures and intermediate state structure decreased, while the crystalline β-sheet content of silk fibroin increased significantly during the gelation process.

**Figure 4:**
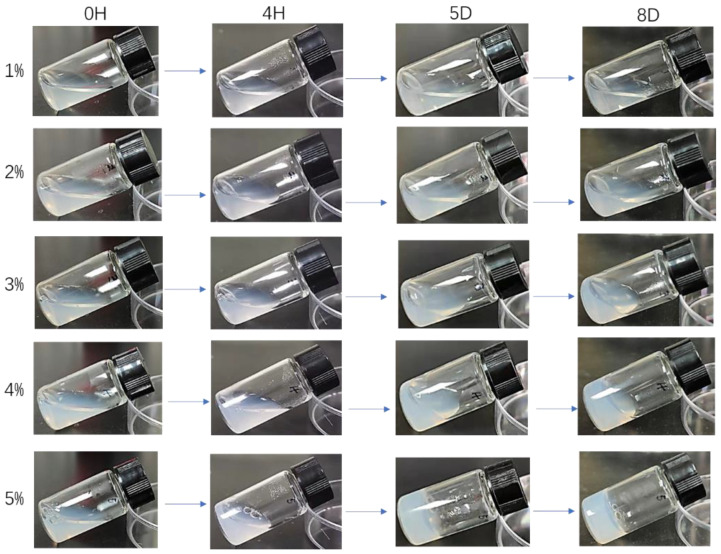
Optical morphology of the CSF solution stored at different time.

The CSF solutions could be stored at 4 °C for 4H-8D or even longer (Figure 4). They reconverted into hydrogels after lengthy storage, and the conversion time decreased with the increase of silk fibroin concentration. The 1% CSF solution didn’t show any obvious change after eight days, while the 5% CSF solution turned into hydrogel after 4 h, and they were easily redistributed. If the solution was placed at room temperature, the time required to convert it into hydrogel would be greatly reduced. This change might be attributable to the instability of high concentration CSF particles in the solution. Silk fibroin molecules could reform hydrogels through self-assembly, and high temperature had a significant role in promoting this process.

### 3.3. Application of Crystalline Silk Fibroin Solution

**Figure 5:**
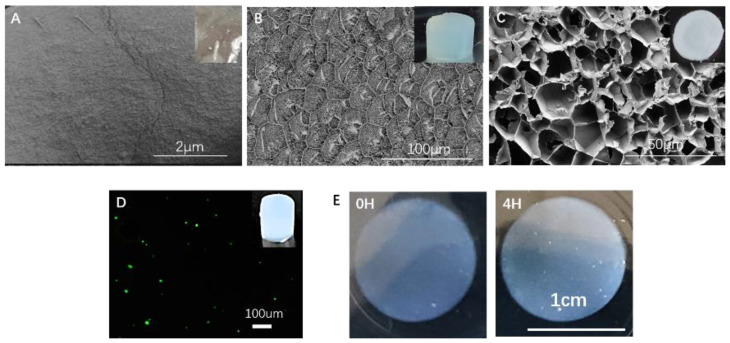
Scaffolds prepared from various silk solutions and gelatin solution. (**A**): SEM of membrane from 3% CSF, (**B**): Morphology of 1% CSF-SF hydrogel, (**C**): Morphology of 6% CSF-SF porous scaffold, (**D**): Cells in 1% CSF-SF hydrogel, (**E**): Morphology of 3% CSF-gelatin hydrogel immersed in PBS at a different time.

Many useful properties of silk fibroin-based materials, such as the unique mechanical properties of natural silk, come from the interaction between ordered and disordered domains. CSF, as an ordered silk fibroin domain, played an important role in the construction of regenerated silk fibroin-based materials. Many kinds of materials, such as membrane, hydrogel and porous scaffold, were constructed based on it (Figure 5). Silk fibroin hydrogel can be constructed by blending CSF solutions with common disordered silk fibroin solution (Figure 5B). Compared with ultrasound-induced hydrogels, this hydrogel has better toughness. Before the gelation, the cell suspension was added into the mixture to obtain the hydrogel which encapsulated the cells. The hydrogel showed good cell compatibility (Figure 5D), while when mixed with the gelatin solution, the CSF solution induced rapid gelation (<1 min) at room temperature, and the silk fibroin/gelatin hydrogel maintained stable morphology at 37 °C without obvious swelling or dissolution (Figure 5E). It provided an excellent choice for the modification of gelatin hydrogel without adding a cross-linking agent. It will be used for neural repair in our future work.

**Figure 6:**
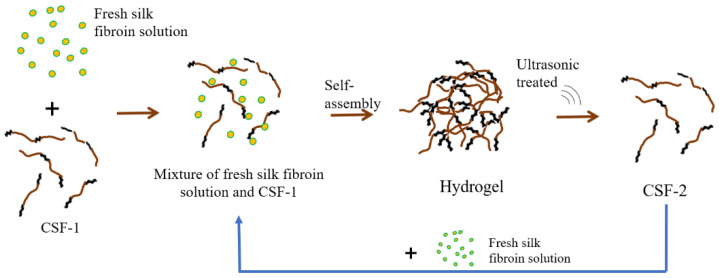
Scheme of the strategy recycling of CSF.

After obtaining silk fibroin hydrogel, CSF solution could be prepared circularly simply by “mix fresh silk fibroin with CSF solution—sonicate the hydrogel” (Figure 6). When the final concentration was low enough, such as 2%, the formed hydrogel could be dispersed by ultrasound to form CSF solution. A new silk fibroin hydrogel could be formed by mixing the CSF solution with fresh silk fibroin solution. The newly formed hydrogel could be dispersed by ultrasound again to form a new CSF. This greatly simplifies the process of preparing the CSF solution.

## 4. Conclusions

In this work, we found a way to prepare crystalline silk fibroin solution by adjusting the silk fibroin solution concentration and the ultrasonic conditions. The solution was composed of β-sheet structure, but remained stable at high concentration. The solution could be mixed with other solutions, such as gelatin and ordinary disordered silk fibroin. Different scaffolds that formed from these solutions, including hydrogels, porous scaffolds and films, showed broad application prospects with useful features for functional materials.

## Figures and Tables

**Table 1:** The relative ratio of secondary structures in solutions or hydrogels.

Assignment	SF Solution	Mixed SF Solution	Flowable Hydrogel	Hydrogel
Side chain (%)	2.0 ± 0.1	1.5 ± 0.3	1.5 ± 0.4	1.5 ± 0.3
β-sheet (%)	21.9 ± 0.5	33.9 ± 0.2	33.9 ± 0.3	48.4 ± 0.3
random coil (%)	33.0 ± 0.5	30.2 ± 0.6	29.5 ± 0.6	24.6 ± 1.0
α-helix (%)	14.8 ± 1.0	13.1 ± 0.6	13.4 ± 0.2	10.3 ± 0.5
β-turn (%)	28.4 ± 2.1	21.3 ± 1.4	21.6 ± 0.8	15.2 ± 1.4

## Data Availability

The data presented in this study are available on request from the corresponding author.

## Supplementary Materials

The following supporting information can be downloaded at: https://www.mdpi.com/article/10.3390/ma15196930/s1, Figure S1: Morphology of silk fibroin hydrogels/solution. (A) Hydrogels before (left) and after (right) ultrasonic with 70% ultrasonic power. After ultrasonic, low concentration hydrogels (1–5%) turned into solutions and high concentration hydrogel (6%) remained solid state. (B) Conversion efficiency of hydrogels with different concentrations under the same ultrasonic power; Figure S2: 5% Hydrogels before (left) and after (right) ultrasonic. With 10% ultrasonic power, only a small amount of gel was converted into solution. With the increase of ultrasonic power, the conversion efficiency increased gradually. When the power was 70%, the conversion efficiency was the highest. When the power reaches 90%, the solution produced decreases; Figure S3: SEM morphology of hydrogels with different concentration.
