# Supplementary material for: High Concentration Crystalline Silk Fibroin Solution for Silk-Based Materials
Source: Materials (Basel). 2022 Oct 6;15(19):6930. doi: 10.3390/ma15196930 (PMC9571966; doi:10.3390/ma15196930)
Supplement: Supplementary file 1 [file materials-15-06930-s001.zip › materials-1904830-supplementary.pdf]

High concentration crystalline silk fibroin solution for silk-based materials

Danyu Yao[1*], Ting Wang [1], Xiaoli Zhang[1,2], Yuqing Wang[3]

1 School of Automation, Hangzhou Dianzi University, Hangzhou 310018, China

2 School of Physics, Huazhong University of Science and Technology,

Wuhan 430074, China

3 Key Laboratory for Biomechanics and Mechanobiology (Beihang University) of Ministry of Education, Beijing Advanced Innovation Center for Biomedical Engineering, School of Biological Science and Medical Engineering, Beihang University, Beijing 100083, China

*Corresponding author: Danyu Yao

E-mail: yaodanyu@hdu.edu.cn

Address: School of Automation, Hangzhou Dianzi University,
Hangzhou 310018, China.

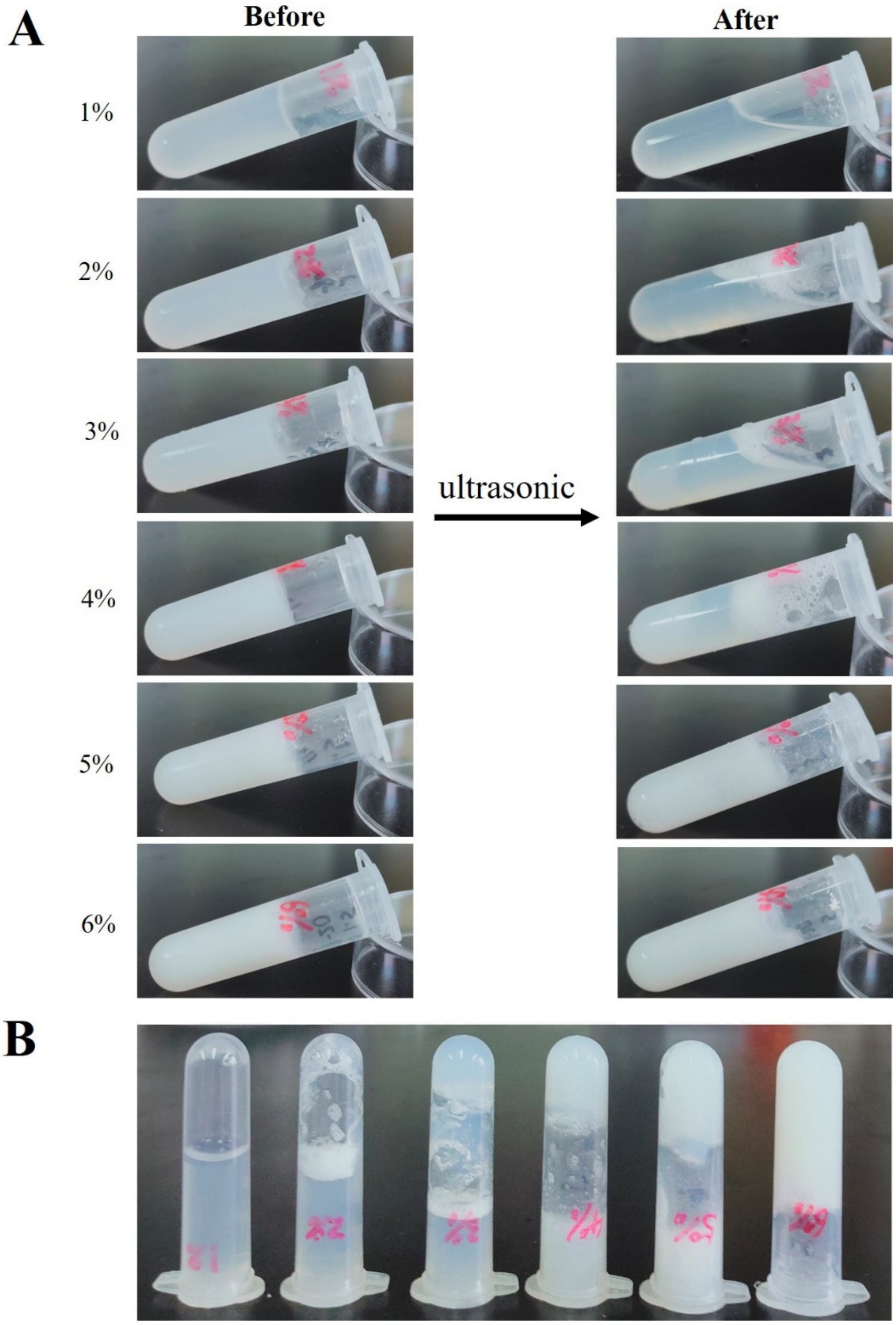

Figure S1: Morphology of silk fibroin hydrogels/solution. A) Hydrogels before (left) and after (right) ultrasonic with 70% ultrasonic power. After ultrasonic, low concentration hydrogels (1-5%) turned into solutions and high concentration hydrogel (6%) remained solid state. B) Conversion efficiency of hydrogels with different concentrations under the same ultrasonic power.

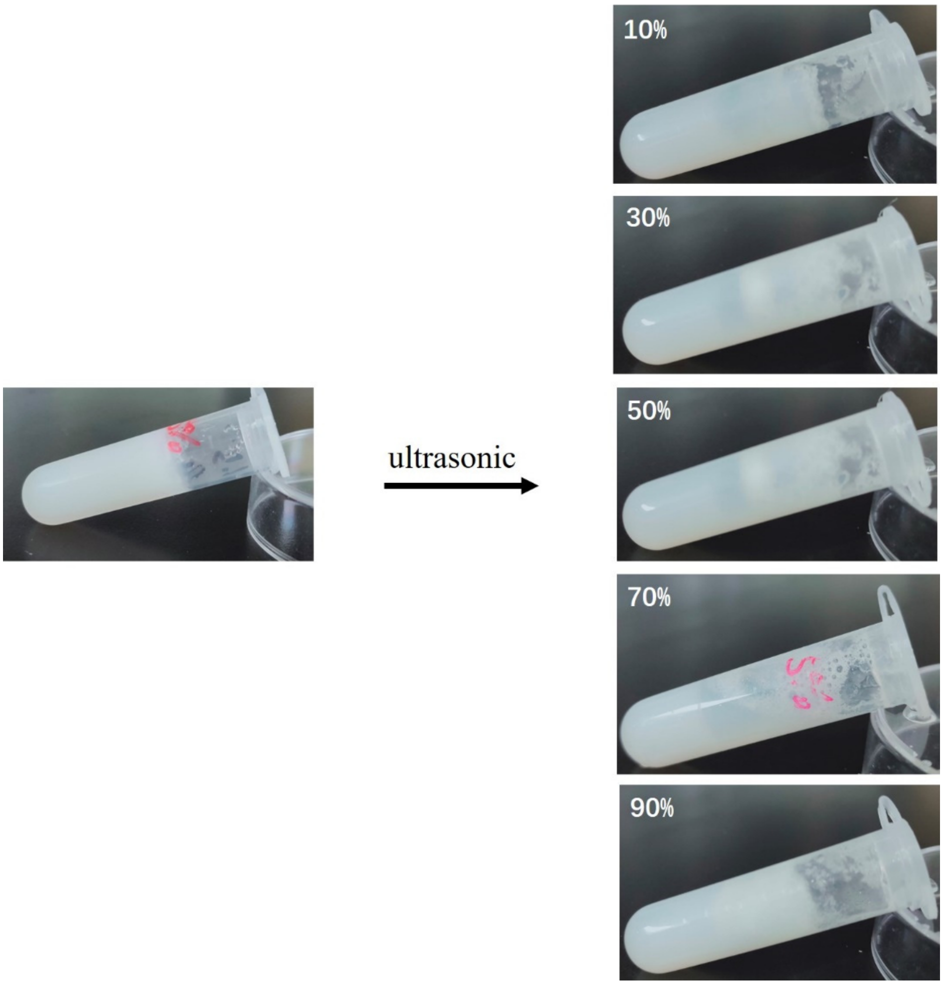

Figure S2: 5% Hydrogels before (left) and after (right) ultrasonic. With 10% ultrasonic power, only a small amount of gel was converted into solution. With the increase of ultrasonic power, the conversion efficiency increased gradually. When the power was 70%, the conversion efficiency was the highest. When the power reaches 90%, the solution produced decreases.

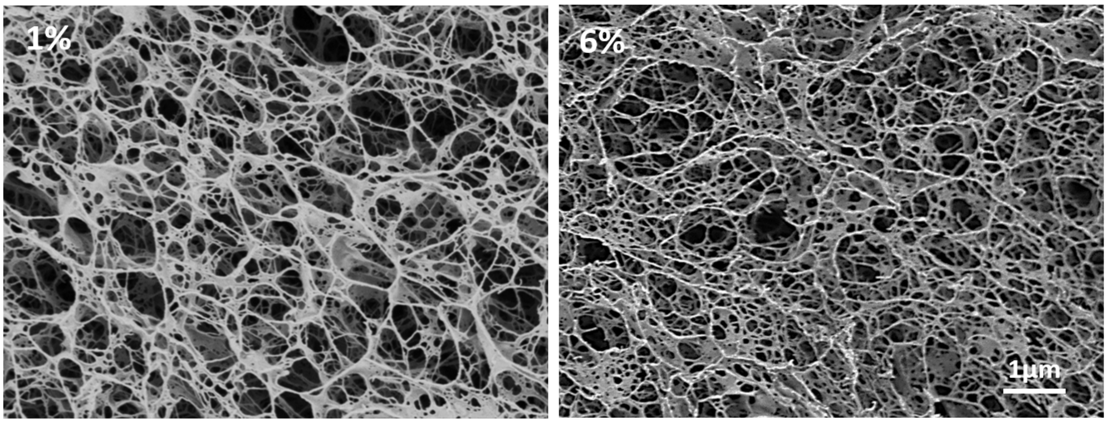

Figure S3: SEM morphology of hydrogels with different concentration.
